# What drives small-scale farmers to vaccinate their multiple livestock species animals against common infectious diseases in Myanmar?

**DOI:** 10.1371/journal.pone.0258765

**Published:** 2021-10-20

**Authors:** Tu Tu Zaw Win, Angus Campbell, Ricardo J. Soares Magalhaes, Kyaw Naing Oo, Joerg Henning

**Affiliations:** 1 The School of Veterinary Science, The University of Queensland, Gatton, Australia; 2 Faculty of Veterinary & Agricultural Sciences, The University of Melbourne, Melbourne, Australia; 3 Children’s Health and Environment Program, The University of Queensland, The University of Queensland, South Brisbane, QLD, Australia; 4 Livestock Breeding and Veterinary Department, The Ministry of Agriculture, Livestock and Irrigation, The Republic of the Union of Myanmar, Yangon, Myanmar; 5 The School of Veterinary and Life Sciences, College of Veterinary Medicine, Murdoch University, Perth, Western Australia, Australia; Universidade do Porto Instituto de Biologia Molecular e Celular, PORTUGAL

## Abstract

Livestock rearing is an important income source for small-scale farmers in Myanmar, but Foot and Mouth Disease (FMD) and Newcastle disease (ND) are major constraints to livestock production. A study was conducted to identify perceptions of farmers about FMD and ND disease risks and perceptions about vaccination practices by using the modified health belief model. The majority of livestock farmers (>70%) reported that they were aware of the risk and impact of FMD and ND and were willing to vaccinate their livestock (>60%). Focusing on three main livestock farmer groups, about 17.0% of cattle, 15.4% of village chicken, but only 2.3% of small ruminant owners, indicated that the non-availability of vaccinations in the villages was the major constraint to vaccinations (p<0.001), while in contrast twice as many small ruminant farmers compared to cattle and village chicken farmers indicated they had no knowledge about vaccinations and no funds to conduct vaccinations. Limited accessibility to vaccines and vaccinators was related to size of villages (p = 0.001 for cattle; p = 0.027 for small ruminants; p = 0.005 for village chicken). Willingness to vaccinate small ruminants against FMD was associated with the perceived impact of the disease on sales and accessibility of information about vaccination. Accessibility to information about ND vaccination influenced the willingness of village chicken farmers to conduct vaccinations. In addition, beliefs in the effectiveness of vaccinations played a major role in the willingness to carry out vaccinations on both, cattle (β = 0.3, p = 0.018) and village chicken farms (β = 0.5, p<0.001). Our study highlights that policies that increase the accessibility of vaccines and the dissemination of information about disease prevention and vaccination practices in villages of all sizes, have the potential to increase FMD and ND vaccination rates and thereby reduce outbreak occurrence in Myanmar. On the other hand, indirect factors, such as village size strongly influenced the availability of vaccinations.

## Introduction

Multispecies, small-scale livestock rearing is the main form of livestock production in low and middle-income countries [1–6]. In multispecies households, cattle are predominately raised for land preparation, while small ruminants are sold for meat and village chickens usually provide supplementary income or eggs and animal protein for consumption [6–10]. Livestock diseases are a major threat to the livelihoods of these small-scale farmers especially in developing countries, but they are also impacting on national food security, economic and social development.

Foot and mouth disease (FMD) is the globally recognized animal disease affecting cattle, swine, sheep, goats and other cloven-hoofed ruminants and causing negative economic impact in animal trading due to its clinical condition, and mortality. With seven different strains (A, O, C, SAT1, SAT2, SAT3, and Asia1), the severity of the disease could be varied depending on strain of virus. In addition, the types of species, the exposure dose, the age and species of animal and the host immunity. Even though the morbidity can reach 100% in susceptible populations, mortality is generally low in adult animals (1–5%), while higher in young calves, lambs and piglets (20% or higher). Chronically affected animals are reported to have an overall reduction of 80% in milk yield. [1,11]. FMD results in reduced efficiency of cattle used for draught power and reduced reproductive performance in cattle and intensively reared goats. Apart from these issue in cattle, the mild or inapparent clinical signs are noticed in sheep and goats[12–16]. Since animal movement is one of the noticeable risk factors for FMD spread, it may further result in trade restrictions on the national and international level [13,15,17–21]. Even though the vaccine with high efficacy is available, the different types of strains (i.e. serotype Asia 1, O/ME-SA/Ind-2001d, and O/SEA/Mya-98) could be challenging for disease control and which may further need for vaccine matching with local strain [17–19,22,23]. The most prevalent FMDV strains are serotype O and A whereas serotype Asia 1 was newly detected in Myanmar in 2005 which could not be clearly explained by the source of origin; and since the dynamic of disease transmission is so complicated, vaccine matching is another challenging for disease control by vaccination for low-income country with limited resource like Myanmar [16,22,24–27]. Due to the awareness, limited resources and vaccination practice especially in rural areas where the majority of traditional backyard farming is implemented, FMD has been still threatening the livestock production especially in developing countries [28,29]. Another high impact disease, which infect poultry is Newcastle disease (ND), with approximately 100% mortality, which is associated with high mortality rates in village chickens and often results in the complete loss of village chicken flocks [21,30–32]. Both of the diseases (i.e. FMD and ND) are recognized globally as high impact notorious endemic diseases in Asia, Africa and Middle East, causing negative impact in the livestock production with its severe clinical signs, reduced performance and slow growth or deaths [11,18,33–37].

Vaccination is an important method for preventing and controlling FMD and ND in livestock [15,38]. In developing countries, vaccination is usually conducted by veterinarians or para-veterinarians employed through the national government veterinary services [39]. Ultimately, livestock farmers decide if their livestock should be vaccinated. Major factors that might influence farmers’ decisions whether or not to vaccinate include farmers’ previous experience with the disease occurrence, social pressure, awareness of the benefits of vaccination, accessibility to information about vaccination, resources to conduct vaccination, accessibility to effective and safe vaccination, the role of regulatory framework and legislation, and personal motivations, but demographics such as gender, age, and socioeconomic status also play a part [40–47]. Understanding attitudes and beliefs about vaccinations as well as barriers for vaccination are important to develop efficient and sustainable disease control strategies. However, it is not fully understood what influences vaccination practices of small-holder farmers in developing countries, in particular on multispecies rearing farms.

Various approaches can be used to study attitudes, perceptions and behaviours [48,49]. One of the them is the Health Belief Model (HBM) framework, which was introduced into health educational research in the 1950s by social psychologists Hochbaum, Rosenstock, and Kegels [50,51]. Since then, the HBM framework has been widely used by researchers in the health psychology to explore the relationship between human cognitive behaviour and health preventive measures, in particular the psychological influences on taking preventive actions to improve human health [52–54]. HBM was developed to understand the effect of the perception on susceptibility, severity of diseases and the benefit of practicing preventive measures which may promote the willingness to practice preventive measures. From the HBM, we try to understand the factors promoting or reducing the willingness or self-efficacy for health practice [51,55,56].

However, the HBM framework has not been widely used to research preventive veterinary actions. We used the HBM framework to investigate the relationship between the perceptions of livestock farmers on barriers and benefits of FMD and ND vaccination and their willingness to practise vaccination against FMD in cattle and small ruminants and ND in village chickens.

## Material and methods

### Study design, sample size and selection of sampling units

This cross-sectional study was conducted with small-scale farmers in two administrative areas in the Central Dry Zone (CDZ) of Myanmar, the Myingyan and Meikhtila Township, which were identified as representative of typical livestock production systems in the CDZ by a research-for-development project investigating livestock production [3]. Subjects for the HBM questionnaire were drawn from a larger sample of households that were surveyed about their cattle, small ruminant and chicken ownership and production. To observe the situation of farming practices in the villages within the two townships, a two-stage sampling approach was used with villages being the primary sampling units (PSUs) and farms the secondary sampling units (SSUs) [57–59]. Sample size was based on the expected proportion of farm income that was generated from livestock production (i.e. 70% of total income was generated from livestock production). The proportion of farm income that was generated from livestock production was expected to be 0.7, with a moderate variation of farm income from livestock production within villages of 0.1 (due to similar ecological conditions), a between cluster variance (between villages variance) of 0.025. Precision of the estimate was set to 0.05 with 95% confidence interval. The number of villages per township was 400 and total farms per village was approximately 200. The online calculator Epi Tools was used to estimate the required sample size using the probability proportion to size algorithm [60]. Sample size calculations and random sampling were performed using the Survey Toolbox modules Sample size for 2-stage prevalence survey (http://epitools.ausvet.com.au/content.php?page=SurveyToolbox) [61]. According to the sample size calculation, a total of 40 villages and 20 farms per village needed to be surveyed. Taking account of the sample size needed to be collected, we selected seven households in each livestock ownership group (cattle, small ruminants, village chickens), thus a total of 21 households per village were selected using simple random sampling from a list of village households. Thereby, 280 farmers of each livestock ownership groups (cattle, small ruminant and village chicken), and a total of 840 households for this study, were subsequently targeted for follow-up interviews on their attitudes towards and practice of FMD and ND vaccination. However, due to the overlapping between the farmers rearing different livestock species, the total of 328 cattle farmers, 3030 small ruminants and 327 village chicken farmers were interviewed. During the data collection, the list of villages and households rearing different livestock species were provided by the Livestock Breeding and Veterinary Department (LBVD) Myanmar.

### Questionnaire and data collection

The HBM questionnaire (including 13 questions relating to each livestock species) was firstly developed in English and then translated into Myanmar (Burmese) language. The questionnaire captured data on demographics, disease prevention practices, individual farmer’s perception on FMD and ND, the effectiveness of and barriers to vaccination and various factors that could impact the likelihood of farmers to have their livestock vaccinated. To test and validate if the questions were applicable to use in the field survey, pilot testing was conducted to local experts included seven members of local authorities, three animal health workers and two research officers. After the pilot testing, some items were modified in the questionnaire. A questionnaire was developed in English and was then translated into the local language (Myanmar) which further used for data collection. Then, the survey was conducted by seven trained interviewers comprising of two veterinary medicine students from the University of Veterinary Science, Yezin, four staff from LBVD and the lead author of this paper. Total interviewing time was approximately 20 minutes for each interview.

### Ethical statement

The study was approved by the University of Queensland Human Research Ethics Committee (approval number #2014001425) and for the local research approval, the approval was obtained from the Livestock Breeding and Veterinary Department (LBVD).

### HBM framework

We used a modified HBM framework to summarize the perceptions of farmers on their willingness to implement vaccinations against FMD and ND. Some questions on HBM components were open-ended (i.e. perceived benefits and cues to action) and were categorized or converted into multiple dichotomized (yes/no) variables for further analysis. We assumed that the farmers (4.6% of cattle farmers; 2.8% of small ruminant farmers; 3.0% of village chicken farmers) who reported ‘don’t know’ to some HBM components were likely to be unaware of the particular item and included these ‘don’t know’ answers in the ‘no’ category. The following modified HBM components were utilized in this study:

1. ***Knowledge about disease*:** Ability of farmers to recognize clinical signs for FMD in ruminants and for ND in village chickens (yes/no). Triangulation to identify farmers’ ability to recognize FMD and ND was done by asking clinical signs, and host.
2. ***Perceived severity (the impact of the disease)*:** Perception of farmers that occurrence of FMD and ND can result in economic losses (i.e. reduced sales or reduced sale prices or unwillingness of traders to purchase disease animals) (yes/no).
3. ***Perceived benefits (the effectiveness of the vaccination)*:** Perception of farmers that FMD and ND vaccination can prevent the occurrence of FMD and ND (yes/no).
4. ***Perceived barriers (the barriers to vaccination)*:** Perceived barriers to conduct FMD and ND vaccinations were categorised into three groups: farmers’ knowledge about the use of vaccination to control FMD and ND (yes/no), availability of vaccination in the village (yes/no) and farmer’s access to funds to pay for vaccination (yes/no).
5. ***Cue to action (the availability of information about vaccination)*:** Accessibility of information on FMD and ND vaccination and vaccination programmes was categorised into four groups: availability of information about vaccination (yes/no), provision of information about vaccination through veterinary administrative officers, local veterinarians and veterinary animal health workers (yes/no); provision of information about vaccination through other farmers (yes/no); and provision of information about vaccination through traders (yes/no).
6. ***Perceived susceptibility (the perception of farmers on the susceptibility of animal to the disease)*:** All of the farmers agreed that their livestock were highly susceptible to the diseases (i.e. FMD and ND). Therefore, we did not take into account this variable for further analysis.

The willingness of farmers to have their animals vaccinated against FMD or ND was used as the outcome variable (yes/no).

We also collected data on factors that could have impacted on HBM components, such as village size, demographic information of farmers (median age: ≤47 years old and >47 years old; gender: male and female; duration of livestock rearing: ≤5 years and >5years), type of animal species reared: raising single species only (cattle/ small ruminant/ village chicken) or combinations), farm income (less than or equal to, or greater than the total median household income of USD 1400 per year); village size (less than and equal to, or greater than total median household number of 188); major income source (cropping, livestock sale, labour, trade and support by relatives) and previous occurrence of clinical FMD and ND on farms (yes/no).

### Statistical analysis

A two-step approach was used to analyse the data: 1) initially descriptive statistics were produced to compare the proportion of farmers holding different perceptions on FMD and ND vaccination between livestock ownership groups; 2) then path analysis was used to investigate the causal factors influencing the willingness of farmers to conduct FMD and ND vaccination for each livestock ownership group.

All data analysis was conducted in STATA 14.0 (Stata Statistical Software, College Station, Stata Corporation, 2015) using a survey design approach by specifying PSU and SSU, sampling weights, sampling strata (townships), clustering (villages) and a finite population correction [62]. Using a survey design approach ensured that correct standard errors were estimated [63–65]. Survey responses were first cross-tabulated and compared between livestock ownership groups. Pearson χ^2^ statistics were converted into F statistics and standard errors and p-value were adjusted to the survey design [66,67]. Binomial logistic regression was used to describe the relationship between the demographic information (age, sex and experience of framers) and the knowledge of farmers on diseases (FMD and ND).

#### Details on path analysis modelling approach

Path analysis is based on multiple regression models that are used to identify the correlation between the exogenous variables representing the variables which are not causally dependent on any other variables, endogenous variables representing the outcome variables explained by the model and endogenous mediator variables representing the variables which intervene between exogenous variable and endogenous outcome variables [68–70]. We used path analysis to identify the relationship between the perceptions of livestock farmers on the severity of FMD and ND, on the barriers and benefits of FMD and ND vaccination, the availability of information about vaccination to farmers, and the outcome of farmers’ willingness to practise vaccination against FMD in cattle and small ruminants and ND in village chickens. Thus, we developed three different models separately for each livestock species: FMD vaccination on any farm owning cattle, FMD vaccination on any small ruminant-owning farm, and ND vaccination to any farm owning village chicken.

**Fig 1:**
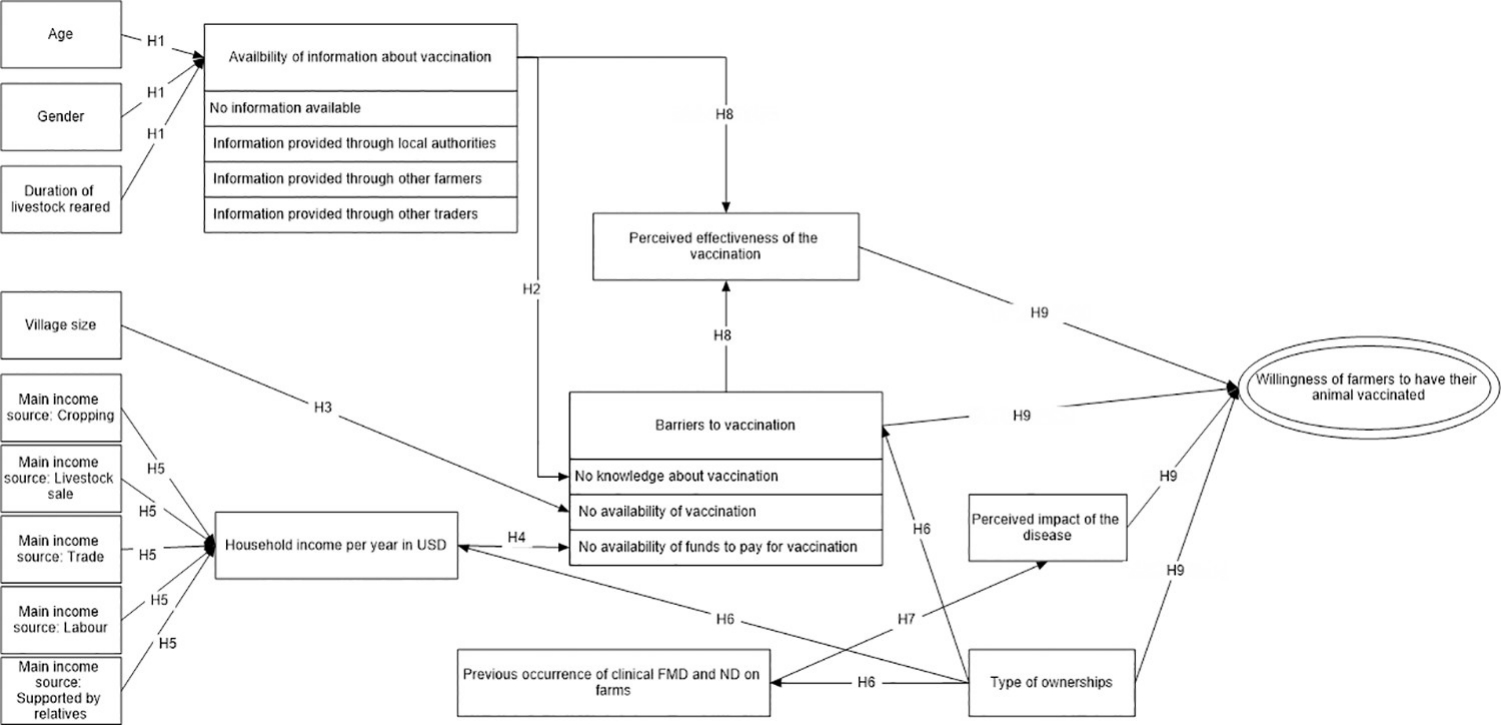
Hypothesized causal diagram to understand the perception of farmers rearing one species on vaccination practice.

First, hypothesized pathways assuming causal relationships between exogenous variables and endogenous variables were developed. Hypothesized causal pathways focussed on nine hypotheses (Fig 1):

H1: Information availability (cues to action) may be associated with age, gender and duration of livestock reared.
H2: Information availability (cues to action) such as no information available about vaccination, information about vaccination provided through local authorities or other farmers or traders may be associated with knowledge of farmers about vaccination.
H3: Availability of vaccination may be associated with village size, due to factors such as infrastructure availability and likely contact with animal health services within or outside the village.
H4: Availability of funds to pay for vaccination may be associated with total household income.
H5: Major income source, such as cropping, livestock sale, labour, trade and supported by relatives, may be associated with household income.
H6: Household income per year in USD, barriers to vaccination and previous occurrence of clinical FMD and ND on farms may be influenced by the type of livestock ownerships (rearing single livestock species or with other species).
H7: Previous occurrence of clinical FMD and ND on farms may also influence the perceived impact of the disease.
H8: Perceived effectiveness of the vaccination may be associated with barriers to vaccination such as knowledge about vaccination, availability of vaccination and information availability about vaccination.
H9: Willingness of farmers to have their animals vaccinated may be predicted by perceived effectiveness of the vaccination, barriers to vaccination, perceived impact of the disease and type of different livestock ownerships (rearing single livestock species or with other species)

To inform the model building, we estimated tetrachoric correlation coefficients for all dichotomous variables for each livestock species separately and variables with significant correlation (p<0.05) were selected for the path analysis for each livestock species (i.e. some hypothetical pathways were removed). We used survey design approaches in the path analysis to account for primary sampling units (PSUs), secondary sampling units (SSUs) and sampling weights.

Path coefficients (also called standardized regression coefficient (beta)) were produced for direct, indirect and total effects. Direct effects represent the effect of one exogenous variable on an endogenous variable. Indirect effects represent the effect of one variable on another variable and thereby making changes to a third variable. Total effects are the sum of direct and indirect effects [68,71,72]. Only responses from farmers who stated that they were able to recognize FMD or ND were used in the path analysis. The fit of the path models was evaluated using standardized root mean squared residuals (SRMR), the coefficient of determination (CD) and the R-squared [73]. In this paper, we presented two figures for each livestock species:—one was the raw model output of the analysis and the other was the clarification of the model output with results.

## Results

### Demographic information of farmers

During the interview, the selection of households was done by simple random sampling and interview was conducted with the family member of the households who volunteer to participate. Therefore, the role of interviewees in the household, participated in this survey, were varied: person taking leading role in the household, person taking care of the animals, and person who worked with cattle for transportation and/or draught purpose. We explored what demographic factors of farmers (age, gender, experience of rearing animals and type of ownerships) influenced the ability of farmers to recognize clinical signs for FMD and ND (Table 1). From our data, we noticed that small ruminant production was more business-oriented farming activity (~41%) which contribute the high proportion of income from livestock sale compared to two other livestock species. Regarding the experience of livestock rearing, experience did not seem to have significant varied in small ruminant production while majority of farmers raising cattle and village chicken had more experience (>5 years). These two facts may explain that the small ruminant market seem to be recently developed which may further lead to raise small ruminant for business-oriented purpose while cattle and village chicken farmers followed traditional farming practice (Table 1).

**Table 1:** Frequency of demographic and farm details of farmers raising cattle, small ruminant or village chickens (Farmer aware of the diseases (FMD and ND) only) and village details.

Modifying factors	Details of survey questions	Categories	Proportion of farmer			F-statistics (p-value)
			Cattle farmer *(N = 366)*	Small ruminant farmer *(N = 252)*	Village chicken farmer *(N = 273)*	
Demographic information	*Gender of farmer*: By observation	Male	52.5 (44.4–60.5)	45.2 (37.3–53.3)	50.7 (43.1–58.3)	2.3 (p = 0.11)
		Female	47.5 (39.5–55.6)	54.8 (46.7–62.7)	49.3 (41.7–56.9)	
	*Age of farmer*: Median value (47 years old) was used as cut-off point	Below median	47.2 (39.1–55.4)	53.3 (43.5–62.9)	50.8 (43.4–58.2)	1.5 (p = 0.24)
		Above median	52.8 (44.6–60.9)	46.7 (37.1–56.5)	49.2 (41.8–56.6)	
	*Duration of livestock reared;* Median value (5 years) was used as cut-off point	Less experience (≤5 years)	8.1 (5.2–12.5)	Goat: 51.2 (42.0–60.3) Sheep: 85.4 (75.3–91.8)	25.8 (18.5–34.8)	31.9^a^ (p<0.001)
		More experience (>5 years)	91.9 (87.5–94.8)	Goat: 48.8 (39.7–58.0) Sheep: 14.6 (8.2–24.8)	74.2 (65.2–81.5)	
Village details	*Village size*: Median value was used as cut-off point	≤188 hh	34.7 (22.8–48.9)	34.5 (22.4–49.0)	35.7 (23.5–50.1)	0.1 (p = 0.87)
		>188 hh	65.3 (51.1–77.2)	65.6 (51.0–77.7)	64.3 (49.9–76.5)	
Household incomes	*Total income per year in USD*: How much money did your household earn over the last 12 months?: Median value across all the farms was used as cut-off point	≤1400 USD per year	44.0 (36.7–51.7)	46.4 (38.0–54.9)	44.0 (35.0–53.3)	0.4 (p = 0.65)
		>1400 USD per year	56.0 (48.4–63.3)	53.6 (45.1–62.0)	56.0 (46.7–65.0)	
	*Major income source*: Which of the following businesses contribute the largest amount of money to your household in a typical year? (Each type was dichotomised in the analysis)	Cropping	53.3 (46.1–60.4)	27.8 (21.1–35.7)	39.6 (31.3–48.5)	7.5^a^ (p<0.001)
		Livestock sale	19.1 (14.3–25.0)	40.9 (32.5–49.8)	25.5 (17.9–35.0)	
		Labour	11.8 (6.4–20.8)	13.3 (7.0–23.8)	14.8 (8.7–24.1)	
		Trade	3.9 (2.1–7.1)	7.7 (4.3–13.2)	7.4 (4.3–12.7)	
		Support by relatives	12.0 (7.6–18.3)	10.4 (5.8–17.7)	12.7 (7.3–21.0)	

(^a^ = p<0.05 in F-statistics).

### Ability of farmers to recognize clinical signs for FMD and ND and their willingness to vaccinate against both diseases

The majority of ruminant farmers (cattle farmers: 95.8% of 328; small ruminant farmers: 80.1% of 303) and village chicken farmers (81.8% of 327) believed they were able to recognize clinical signs for FMD in ruminants and for ND in village chickens.

Male farmers rearing cattle were 14.6 times (95%CI: 1.6–130.8, p = 0.018) more likely to report they could recognize clinical signs of FMD than female farmers. No association between gender and ability to recognize FMD or ND signs was found for small ruminant and village chicken farmers. Other factors such as age, experience and type of ownership were not associated with recognizing clinical signs of FMD and ND.

Amongst only farmers who reported being able to recognize FMD or ND signs, the willingness to practise vaccinations differed between the three main livestock farmer groups (p<0.001), with 88.0% of cattle farmers, 83.9% of small-ruminant farmers and 71.3% of village chicken farmers being willing to vaccinate their animals (Table 2).

**Table 2:** Frequency of perceptions and practices of FMD or ND vaccination amongst farmers raising cattle, small ruminant or village chickens (Farmer aware of the diseases (FMD and ND) only).

HBM components and modifying factors	Details of survey questions	Categories	Proportion of farmer			F-statistics (p-value)
			Cattle farmer *(N = 366)*	Small ruminant farmer *(N = 252)*	Village chicken farmer *(N = 273)*	
Previous occurrence of clinical FMD and ND on farms	Have you seen the following clinical signs in your farm? Dichotomized for each category	Sore or abnormal hoof, foot or leg causing abnormal movement and other physical abnormalities (i.e. FMD signs) for cattle and small ruminant; and twisted head and neck and other physical abnormalities (i.e. ND signs) for village chicken	21.2 (16.7–26.5)	39.8 (30.6–49.7)	35.7 (27.7–44.7)	8.8^a^ (p<0.001)
Perceived severity	***Perceived impact of the diseases*:** Do you think the incidence of the disease* in your farm animals can cause loss in marketing and trading (i.e. reduce sale or sale prices or traders are not willing to buy animals)?	FMD for cattle and small ruminant; and ND for village chicken	75.2 (69.1–80.4)	81.0 (74.8–86.0)	91.5 (85.6–95.1)	11.1^a^ (p<0.001)
Perceived effectiveness	***Perceived effectiveness of vaccination*:** Do you think that the vaccination** can prevent the following disease* occurrence? (Dichotomized for each categories)	FMD for cattle and small ruminant; and ND for village chicken	83.2 (78.1–87.4)	83.0 (77.0–87.7)	72.8 (65.6–78.9)	1.7 (p = 0.19)
Perceived barrier	***Barriers to vaccination*:** What are the main barriers or obstacles to conduct vaccination**? (Dichotomized for each categories)	No availability of fund to pay for vaccination	9.1 (6.7–12.3)	12.9 (9.0–18.1)	6.6 (3.7–11.6)	4.2^a^ (p<0.05)
		No knowledge about vaccination	7.1 (4.3–11.7)	18.7 (13.4–25.7)	5.3 (3.0–9.2)	16.3^a^ (p<0.001)
		No availability of vaccination	17.5 (12.6–23.7)	2.3 (0.9–5.7)	15.4 (10.4–22.0)	26.1^a^ (p<0.001)
Cue to action	***Availability of information about vaccination*:** From whom did you receive some guidance or instructions about vaccination** programme? (Dichotomized for each categories)	No information availability	19.4 (14.7–25.3)	38.3 (30.8–46.3)	57.7 (50.9–64.3)	38.4^a^ (p<0.001)
		Information provided through local authorities	75.0 (68.0–80.9)	48.0 (38.5–57.6)	35.3 (28.6–42.6)	32.8^a^ (p<0.001)
		Information provided through other farmers	4.3 (2.2–7.9)	6.5 (3.5–11.7)	5.2 (2.8–9.6)	0.98 (p = 0.38)
		Information provided through traders	1.3 (0.5–3.4)	7.3 (4.5–11.7)	1.7 (0.4–6.7)	6.7^a^ (p<0.05)
Likelihood of practicing vaccination	***Willingness of farmers to have their animals vaccinated*:** Would you like to practise the vaccination** in your farm animal? (Dichotomized for each categories)	FMD vaccination	88.0 (81.6–92.4)	83.9 (74.2–90.4)	N/A	10.6^a^ (p<0.001)
		ND vaccination	N/A	N/A	71.3 (64.6–77.2)	

(* = FMD in cattle and small ruminant and ND in village chicken

** = FMD vaccination in cattle and small ruminant and ND vaccination in village chicken

^a^ = p<0.05 in F-statistics).

Focusing the rest of the analysis on the three main livestock farmer groups, there were significant differences in the barriers to practise vaccination, availability of information about disease prevention and vaccination in the villages and previous occurrences of FMD and ND signs (p<0.05). About 17.0% of cattle, 15.4% of village chicken, but only 2.3% of small ruminant owners, indicated that the non-availability of vaccinations in the villages was the major constraint to vaccinations (p<0.001), while in contrast twice as many small ruminant farmers compared to cattle and village chicken farmers indicated they had no knowledge about vaccinations and no funds to conduct vaccinations (Table 2).

About 19.4% of cattle, 38.3% of small ruminant, but 57.7% of village chicken owners indicated that no information is provided to them about the prevention of major infectious diseases (p<0.001). Local authorities were the main provider for information on disease prevention and vaccinations (although less frequent on ND prevention in village chicken), while traders seemed to be an important additional source of information about FMD vaccinations for small ruminant farmers. The proportion of farmers who reported severe impacts of disease on the sale of animals was higher for village chicken (91.5%) and small ruminant farmers (81.0%) compared to cattle farmers (75.2%) (p<0.001). Regarding the impact of disease, small ruminants were raised for more business-oriented purpose and any ulcer or abnormality causing negative impact in animal sale was reported while cattle were raised for long term. Even though the clinical signs were more obvious in cattle, due to low mortality rate, farmers did not seem to consider FMD to be a high impact disease. This is also reflected in small ruminant and village chicken farmers reporting previous occurrence of clinical FMD and ND signs on their farms compared to cattle households (Table 2).

### Factors that influence farmers’ willingness to vaccinate their livestock against FMD and ND

#### Correlations between farmers’ perceptions about FMD and ND vaccinations, types of livestock reared, farmers’ demographics and farmers’ willingness to conduct vaccinations

Tetrachoric correlations between farmers’ perceptions about FMD and ND vaccinations, types of livestock reared, farmers’ demographics and farmers’ willingness to conduct vaccination are shown in S1–S3 Tables. Similar correlations were observed for all three-livestock species: information available through local authorities was negatively correlated with no knowledge about vaccination (r = -0.4, p<0.05 for cattle; r = -0.1, p>0.05 for small ruminants; r = -1.0, p<0.05 for village chicken). No information available was negatively correlated with perceived impact of disease (i.e. FMD for cattle and small ruminants, and ND for village chicken) (r = -0.2, p<0.05 for cattle; r = -0.3, p<0.05 for small ruminants; and r = -0.3, p>0.05 for village chicken) and positively correlated with no knowledge about vaccination (r = 0.5, p<0.05 for cattle; r = 0.03, p>0.05 for small ruminants; r = 0.4, p<0.05 for village chicken). Perceived impact of disease was positively correlated with perceived effectiveness of vaccinations (r = 0.2, p<0.05 for cattle; r = 0.3, p<0.05 for small ruminant; r = 0.5, p<0.05 for village chicken) while no knowledge about vaccination was negatively correlated with perceived effectiveness of vaccinations (r = -0.5, p<0.05 for cattle; r = -0.4, p<0.05 for small ruminant; r = -0.5, p<0.05 for village chicken). Village size was positively correlated with both perceived effectiveness of vaccination (r = 0.3, p<0.05 for cattle; r = 0.2, p>0.05 for small ruminant; r = 0.2, p>0.05 for village chicken) and willingness of farmers to have their animals vaccinated (r = 0.3, p<0.05 for cattle; r = 0.3, p<0.05 for small ruminant; r = 0.2, p>0.05 for village chicken) (S1–S3 Tables).

#### Path analysis modelling to understand factors influencing farmers’ willingness to vaccinate cattle against FMD

Perceived effectiveness of the FMD vaccine was a crucial factor for cattle farmers to implement FMD vaccinations (β = 0.3 [0.1–0.5], p = 0.018), while poor knowledge about the use of vaccinations to control FMD reduced the overall willingness to conduct vaccinations (β = -0.4 [-0.7- -0.2], p = 0.000), but also reduced farmers’ beliefs in the effectiveness of the FMD vaccine (β = -0.2 [-0.4- -0.1], p = 0.009). In addition, an understanding of farmers that FMD can result in severe economic losses increased their belief in the effectiveness of FMD vaccinations (β = 0.1 [0.01–0.3], p = 0.034). As expected, increased availability of information about FMD control increased farmers’ knowledge about the purpose and use of FMD vaccinations (β = 0.2 [0.1–0.3], p = 0.002), while unavailability of vaccination campaigns in a village reduced farmers’ knowledge about the purpose and use of FMD vaccinations (β = 0.1 [0.03–0.1], p = 0.039). Thus, both, the cattle farmers’ knowledge about FMD control (β = -0.4 [-0.7- -0.2], p = 0.000) and the availability of FMD vaccine (β = 0.04 [-0.1–0.2], p = 0.416) are key determinants to improve cattle farmers’ willingness to practise FMD control.

**Fig 2:**
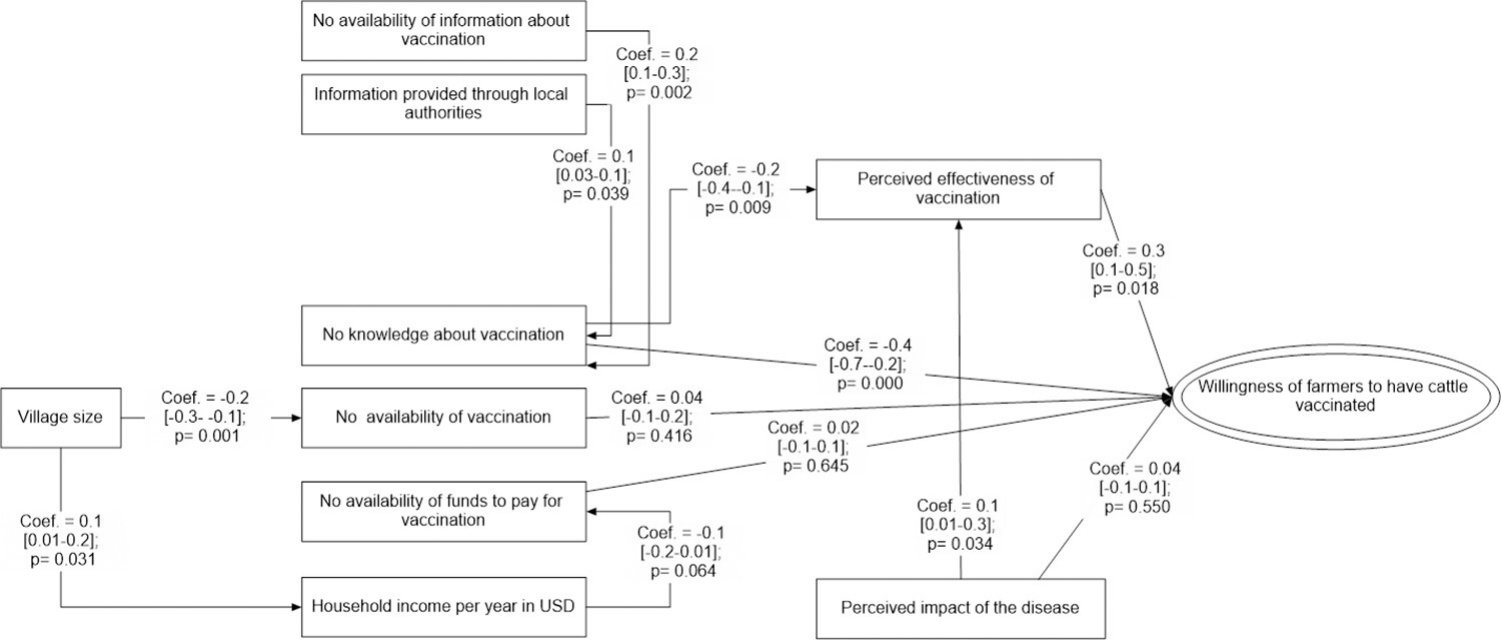
Causal path modelling approach to understand farmers’ perception on Foot and Mouth Disease (FMD) vaccination practice in cattle production indicating Coef: Path coefficient with confident limit; p: p-value.

In larger villages, total income from cattle production was higher (β = 0.1 [0.01–0.2], p = 0.31), resulting in more funds being available (β = -0.1 [-0.2–0.01], p = 0.064) to cattle famers to conduct FMD vaccination, which in turn also positively impacted on the availability of FMD vaccines in villages (β = -0.2 [-0.3- -0.1], p = 0.001). The latter might be a result of cattle farmers with larger incomes ‘requesting’ FMD vaccination campaigns to be conducted in their villages (β = 0.02 [-0.1–0.1], p = 0.645) (Figs 2 and S1).

The final path model describing the perceptions of cattle farmers about having their animals vaccinated had a reasonable fit with a SRMR of 0.043 and CD of 0.122.

#### Path analysis modelling to understand factors influencing farmers’ willingness to vaccinate small ruminant against FMD

The perceived economic impact on sales was the driving factor for small ruminant farmers to implement FMD vaccinations (β = 0.2 [0.1–0.3], p = 0.005), while the non-availability of information about FMD vaccination was the major limiting factor (β = -0.2 [-0.3- -0.03], p = 0.014)).

Similarly, village size had significant indirect impact, as in larger villages greater availability of vaccination was observed (β = -0.1 [-0.1- -0.01], p = 0.027), but also the income of small ruminant farmers was increased (β = 0.2 [0.03–0.3], p = 0.020). No availability of funds to conduct vaccinations also reduced the availability of information about vaccination (β = -0.2 [-0.4- -0.01], p = 0.039). This could be assumed that famers with limited funds were less likely to access information about FMD vaccinations, which in turn they cannot afford FMD vaccination or any preventive actions in general (β = -0.2 [-0.3- -0.03], p = 0.014). The perceived effectiveness of FMD vaccine was not a factor impacting on the willingness of small ruminant farmers to conduct FMD vaccinations.

**Fig 3:**
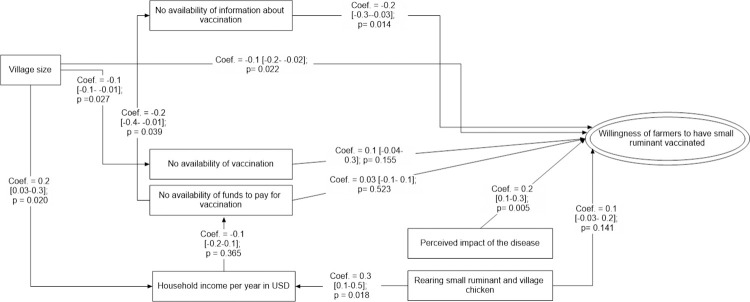
Causal path modelling approach to understand farmers’ perception on Foot and Mouth Disease (FMD) vaccination practice in small ruminant production indicating Coef: Path coefficient with confident limit; p: p-value.

The income measured in this study was based on income within 1 year period which mean income within a short period. According to the farmers, rearing small ruminant and village chicken could help to get income within short period regardless of health problems. This explained by the statistical result: rearing small ruminants together with village chickens increased a small ruminants farmer’s income (β = 0.3 [0.1–0.5], p = 0.018), although the overall impact of raising these two species together on the willingness to conduct FMD vaccinations is unclear (β = 0.1 [-0.03–0.2], p = 0.141) (Figs 3 and S2).

The final path model describing the willingness of small ruminant farmers to have their animals vaccinated had a reasonable fit with a SRMR of 0.049 and CD of 0.187. The modification index suggested to include a path between village size and the willingness to conduct FMD vaccinations in the final path model.

#### Path analysis modelling to understand factors influencing farmers’ willingness to vaccinate village chicken against ND

**Fig 4:**
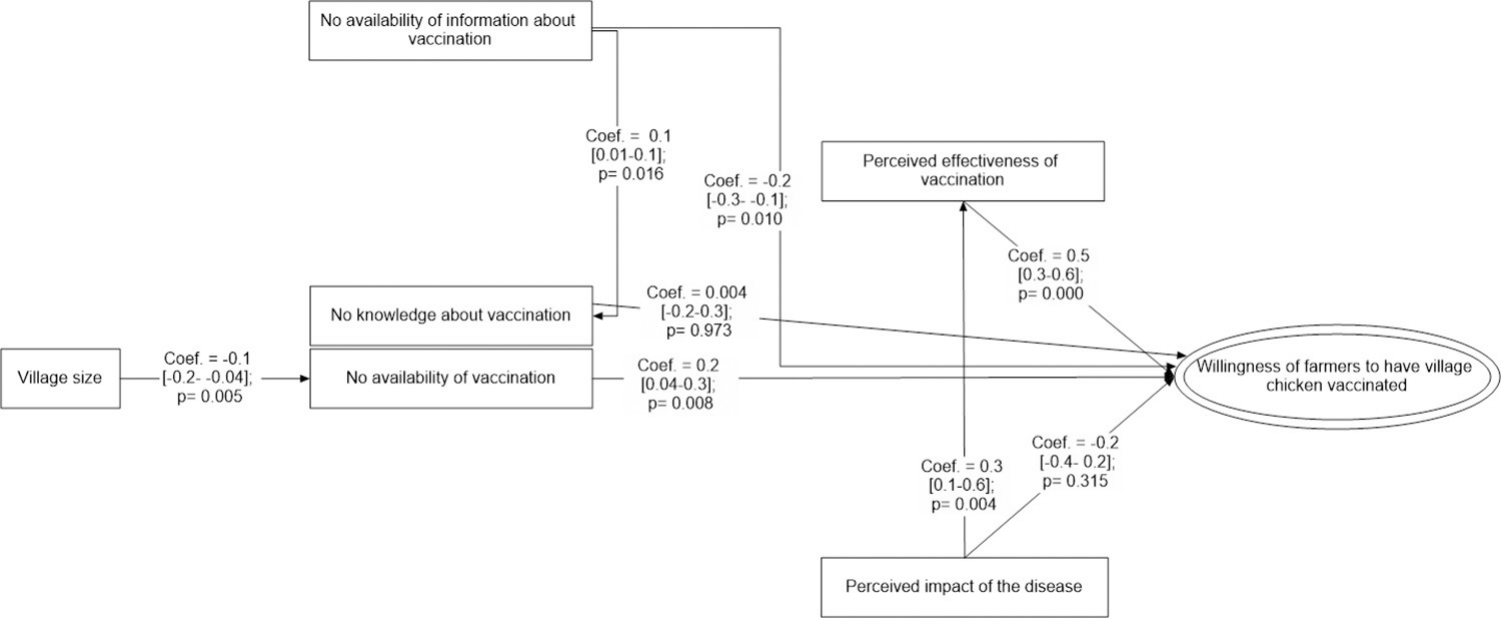
Causal path modelling approach to understand farmers’ perception on Newcastle disease (ND) vaccination practice in village chicken production indicating Coef: path coefficient with confident limit; p: p-value.

Similar to cattle households, the perceived effectiveness of the vaccine (ND) was the driving force for village chicken farmers to implement vaccinations (β = 0.5 [0.3–0.6], p<0.001), while an understanding of the economic losses of ND outbreaks increased farmers beliefs in the effectiveness of the ND vaccine (β = 0.3 [0.1–0.6], p = 0.004). Unavailability of information about ND vaccination reduced willingness of farmers for ND vaccination (β = -0.2 [-0.3- -0.1], p = 0.010), but was also directly related to village chicken farmers’ knowledge about the purpose and use of ND vaccinations (β = 0.1 [0.01–0.1], p = 0.016). And once again, in smaller villages the availability of ND vaccine was limited (β = -0.1 [-0.2- -0.04], p = 0.005) which directly impacted on the willingness of farmers to conduct ND vaccinations (β = 0.2 [0.04–0.3], p = 0.008) (Figs 4 and S3). The final path had a reasonable fit with a SRMR of 0.038 and CD of 0.216.

#### Indirect effects

The final path models revealed similarities of direct effects for the three livestock ownership groups, but also similar indirect effects impacting on the willingness of farmers to vaccinate their animals. For example, perceived impact of the disease based on the economic losses associated with diseases (i.e. FMD and ND) (Indirect effect: β = 0.05, SE = 0.02, p = 0.032 in cattle; β = 0.09, SE = 0.06, p = 0.023 in village chicken), but also unavailability of vaccinations (Indirect effect: β = -0.05, SE = 0.03, p = 0.063 in cattle; β = -0.02, SE = 0.02, p = 0.247 in small ruminants; β = -0.06, SE = 0.07, p = 0.056 in village chickens) indirectly impacted on the willingness of farmers to vaccinate (Tables 3–5).

**Table 3:** Path analysis modelling approach to understand the perception of cattle farmers on FMD vaccination practice.

Variables	Std. coefficient	SE
**Indirect effect**		
***Perceived effectiveness of the vaccination <-***		
No availability of information about vaccination	-0.04*	0.02
***Willingness of farmers to have cattle vaccinated <-***		
Perceived impact of the disease	0.05*	0.02
No availability of information about vaccination	-0.09**	0.03
**Total effects**		
***No knowledge about vaccination <-***		
No availability of information about vaccination	0.25**	0.05
Information provided through local authorities	0.09*	0.02
***No availability of vaccination <-***		
Village size	-0.20**	0.04
***Total income per year in USD <-***		
Village size	0.12*	0.06
***Perceived effectiveness of the vaccination <-***		
No knowledge about vaccination	-0.16**	0.08
Perceived impact of the disease	0.17*	0.07
No availability of information about vaccination	-0.04*	0.02
***Willingness of farmers to have cattle vaccinated <-***		
No knowledge about vaccination	-0.38***	0.10
Perceived effectiveness of the vaccination	0.30*	0.11
No availability of information about vaccination	-0.09**	0.03

(p-value: * = <0.05; ** = <0.01; *** = <0.001).

**Table 4:** Path analysis modelling approach to understand the perception of small ruminant farmers on FMD vaccination practice.

Variables	Std. coefficient	SE
**Total effects**		
***No availability of information about vaccination <-***		
No availability of funds to pay for vaccination	-0.14*	0.09
***Total income per year in USD <-***		
Rearing small ruminant and village chicken	0.21*	0.11
Village size	0.17*	0.07
***Willingness of farmers to have small ruminant vaccinated <-***		
Availability of information about vaccination	-0.20*	0.06
Perceived impact of the disease	0.22**	0.07
Village size	-0.17*	0.05
***No availability of vaccination <-***		
Village size	-0.15*	0.02

(p-value: * = <0.05; ** = <0.01; *** = <0.001).

**Table 5:** Path analysis modelling approach to understand the perception of village chicken farmers on ND vaccination practice.

Variable	Std. coefficient	SE
**Indirect effect**		
***Willingness of farmers to have village chicken vaccinated <-***		
Perceived impact of the disease	0.09*	0.06
**Total effects**		
***Willingness of farmers to have village chicken vaccinated <-***		
Perceived effectiveness of the vaccination	0.44***	0.08
No availability of vaccination	0.12**	0.06
No availability of information about vaccination	-0.29***	0.05
***No knowledge about vaccination <-***		
Availability of information about vaccination	0.15*	0.03
***Perceived effectiveness of the vaccination <-***		
Perceived impact of the disease	0.21**	0.11
***No availability of vaccination <-***		
Village size	-0.19**	0.05

(p-value: * = <0.05; ** = <0.01; *** = <0.001).

## Discussion

In this study, we explored the effects of the perception of livestock farmers on their willingness to conduct FMD vaccinations in cattle and small ruminants and ND vaccination in village chickens. This study is novel in a number of ways. Firstly, data collected focused on the identification of the likelihood of having their livestock vaccinated in multispecies owning households. Secondly, it used the health belief framework to explore factors impacting on willingness to conduct vaccinations while comparing cattle, small ruminant and village chicken households.

There are a number of health behaviour models and methodologies such as Health Belief Model (HBM), the Theory of Reasoned Action (TRA), the Theory of Planned Behaviour (TPB) and the Trans-Theoretical Model (TTM), to observe the drivers triggering health behaviour, we did use HBM in our study. Even though most of the health behaviour model have basic similarity such as consideration on vulnerability of individual, barriers and benefit, health belief model have a number of advantages such as clear identification of the role of demographic variables, the impact of cue to action, and threat on motivation of disease prevention practice and changing behaviour [51,53,55,74,75]. In this study, we use the health belief model (HBM) to describe the farmers’ perception of the severity of FMD and ND, the barriers to practising vaccination, the availability of information about vaccinations, and perceived effectiveness of vaccination. We then identified the factors influencing farmers’ attitudes and awareness towards FMD and ND vaccination practices. This information could help to develop appropriate FMD and ND control strategies considering the perceptions of farmers.

Willingness of farmers to vaccinate their livestock differed between the three major livestock species, with cattle and small ruminant farmers being more willing to vaccinate than village chicken farmers, probably due the different value of livestock species to the household income. Interestingly, keeping combinations of different livestock species, a common feature in small-scale multispecies households in Myanmar, did not impact on the willingness of farmers to vaccinate. For cattle and village chicken owners, the perceived impact of FMD and ND, in particular reduced weight gain, reduced production and mortalities [76,77] was highly recognised by farmers. However, even though sheep and goats are transporter of FMD, the perceived impact of FMD in these species were not highly recognized by farmer as they do not exhibit clear symptoms like cattle and pigs. In addition, the experiences of previous vaccinations, seem to influence their trust in the effectiveness of FMD and ND vaccines and thereby increased their willingness to vaccinate. However, due to the prevalence of different strain of FMDV (i.e. serotype O, A and Asia 1), vaccine matching has also been a challenge for FMD control by vaccination since the FMD vaccine for serotype O and A are mostly available in Myanmar [16,22,24–27]. For poultry, it does not seem to be the case in ND vaccination. In addition to vaccine matching with the field strain, cold chain system, storage and quality of vaccine, nutrition and body condition of animals may affect the farmer experience and perception on effectiveness of vaccination [38,78–87]. Another possible factor triggering the willingness to practice vaccination could be law and regulation of livestock trading and animal movement which was not measured in this study. According to the Animal Health and Development Law issued by Livestock Breeding and Veterinary Department, the cross-border trading of animals with Foot and Mouth Disease are highly restricted and that could affect in legal livestock trading and price of livestock [45,46]. For small ruminant farmers, the perceived economic impact of FMD directly influenced the willingness to vaccinate, probably as the sale of animals is the main reason for raising small ruminants [4] and therefore farmers are very concerned about the impact of FMD on their livestock sales.

Limited availability of information about livestock diseases and their prevention and unavailability of vaccination campaigns were identified as major barriers. However, the availability of information and the vaccine differed between the three livestock species groups, which is a reflection of the limitations of animal health and veterinary services [88,89] and information campaigns to equally cover all livestock species [88,89,89]. Interestingly, public awareness and advocacy was more likely to be accessed for cattle and village poultry farmers while the small ruminant farmers with limited funds were less likely to access information about vaccination practice in small ruminant, which in turn lead to affect willingness of having their animal vaccinated. Surprisingly, about 17.5% of cattle and 15.4% of village chicken owners, but only 2.3% of small ruminant owners indicated non-availability of vaccination affected their willingness to vaccinate. The reason for this might be that small ruminant farmers might actually not be aware of the existence of an FMD vaccine–this is also supported by the observation that twice as many small ruminant farmers compared to cattle and village chicken farmers had no knowledge about vaccinations and no funds to conduct vaccinations. This could be explained by the results of limited information of vaccination and knowledge on vaccination seems to have high influence on the willingness of having their animal practice that was consistent across three different livestock species (i.e., cattle, small ruminant and village chicken farmers: p<0.05). According to literature, even though gender plays a critical role on decision making due to knowledge, experience, education level, and role in the household, the gender did not seem to significantly influence on willingness of vaccination practice [90–92]. This suggests that raising public awareness and facilitating availability of vaccine matching local strains is critical for promoting the practice of vaccination and vaccine efficacy.

It has been highlighted previously that promoting awareness about infectious livestock diseases will increase vaccination rates [88,89]. However, it is essential to use appropriate extension messages and approaches to advise farmers on methods to improve livestock health [93]. Our study identified that accessibility to information and to vaccinations was determined by village size. Thus, vaccinations and information campaigns were not uniformly conducted in all rural areas and most likely campaigns focused on easily assessable locations or more densely populated areas (which often have a better infrastructure such as roads and therefore can be more easily reached). However, trade of livestock and animal movements are the main factors supporting the spread of FMD and ND viruses between farms, villages and markets [15,16,94] and thus there should be no excuse for smaller villages to be excluded from disease prevention programmes. Supporting both large and small villages in the prevention of infectious ruminant and poultry diseases will help to improve the endemic FMD and ND situation and ultimately to improve the livelihood of farmers. During informal discussions with some cattle farmers, concerns about adverse effects of vaccination such as “cattle becoming dull and insipid to work in the field” or “cattle showing depression after vaccination” were raised–thus, it seems, that larger villages with better access to vaccinations might have experienced unsatisfactory vaccination effects. However, the importance of this observation is not clearly understood and further research study is recommended to investigate.

Our study had a number of limitations. Firstly, responses of farmers to questions using the health belief framework were dichotomised as farmers were unable to provide more detailed answers on a Likert-type scale. Secondly, the two diseases studied here might present themselves by a wide range of clinical symptoms and farmers might not have been able to correctly identify these diseases. Therefore, we focused our analysis only on farmers who were able to recognize FMD and ND symptoms. Thirdly, the sample size calculation used in this study considered the contribution of livestock production on household income and not based on health belief model. Fourth, we did use path analysis to understand and analyze the relationship between variates and co-variates which was estimated by coefficient and the type of data used in this study were dichotomous data. In path analysis, variables were assumed as linear, causal and additives; whereas residuals are not correlated with variables; and variables were measured without error which may affect in the interpretation. For further study, we would like to suggest to test mean-adjusted weighted least squares estimation by using structural equation modelling (SEM). However, using path analysis could also explain the decomposition of correlation between the variables, thereby interpretation of relations as well as the patter of the effects of one variable to another by total effect, direct effect and indirect effects via mediation [95].

## Conclusions

We identified that perceptions on the effectiveness of vaccination, poor knowledge about the use of vaccination and limited availability of vaccine and vaccinators limited the willingness of farmers to conduct vaccinations, while the perceived impact of the diseases increased farmers’ willingness for preventive actions. On the other hand, indirect factors, such as village size strongly influenced the availability of vaccinations. Our study highlights that policies that increase vaccine access and the dissemination of information about disease prevention and vaccination practices in village of all sizes, have the potential to increase FMD and ND vaccination rates and thereby reduce outbreak occurrence. Our results show that village size has a huge impact on vaccine availability, indicating that vaccination practice was not widely available in rural areas where the majority of backyard farming is practiced. This may promote the prolonged existence of endemic diseases within the nation and further extend the spread of these diseases. Based on the findings from the current study, we conclude that promoting public awareness along with facilitating the vaccine availability in both urban and rural area is critically important for the national disease prevention and control strategy.

## Supporting information

S1 Checklist (DOCX)

S1 Fig The complete path model analysis to understand the factors affecting the willingness of farmers to have cattle vaccinated. (TIF)

S2 Fig The complete path model analysis to understand the factors affecting the willingness of farmers to have small ruminant vaccinated. (TIF)

S3 Fig The complete path model analysis to understand the factors affecting the willingness of farmers to have village chicken vaccinated. (TIF)

S1 Table Correlation coefficient of health belief criteria of cattle farmers on FMD vaccination using tetrachoric correlation coefficient (* *p*<0.05). (DOCX)

S2 Table Correlation coefficient of health belief criteria of small ruminant farmers on FMD vaccination using tetrachoric correlation coefficient (* *p<0*.*05*). (DOCX)

S3 Table Correlation coefficient of health belief criteria of village chicken farmers on ND vaccination using tetrachoric correlation coefficient (* *p<*0.05). (DOCX)

S1 File (PDF)
