# Supplementary figures and images for: What drives small-scale farmers to vaccinate their multiple livestock species animals against common infectious diseases in Myanmar?
Source: PLoS One. 2021 Oct 20;16(10):e0258765. doi: 10.1371/journal.pone.0258765 (PMC8528287; doi:10.1371/journal.pone.0258765)

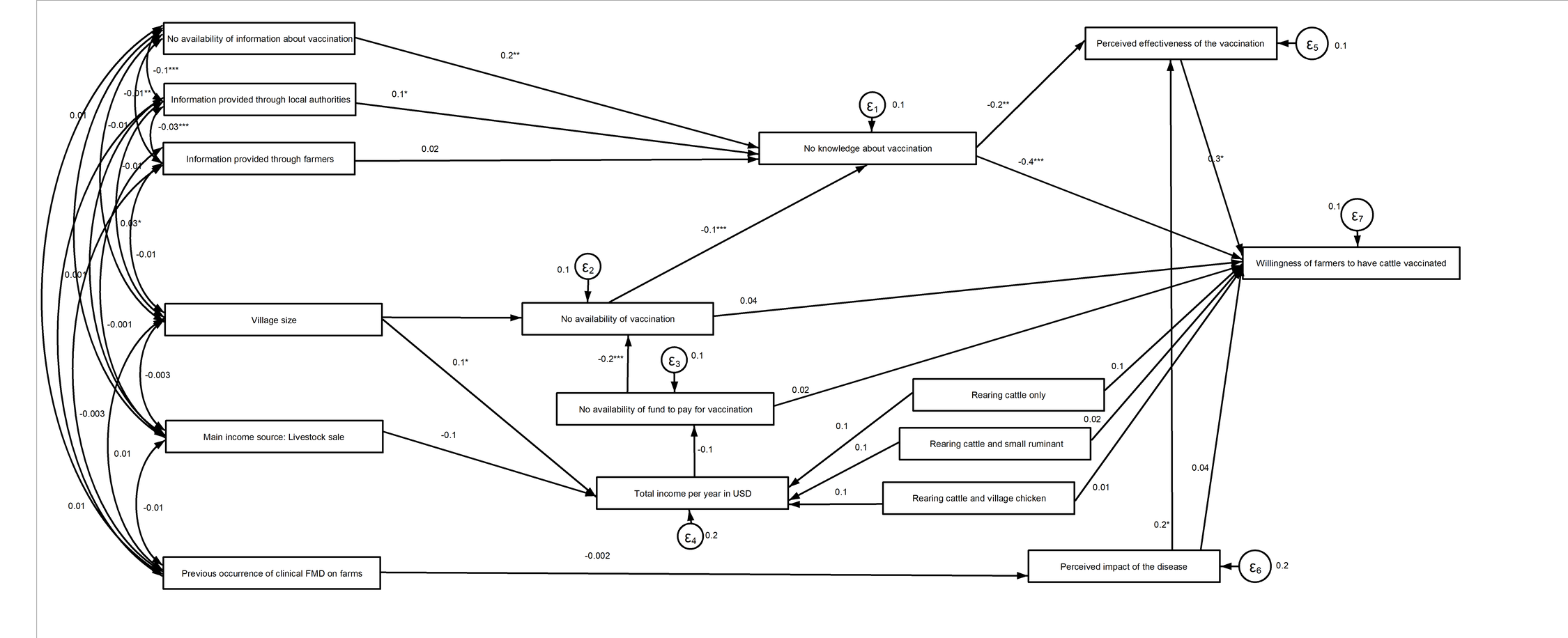

Supplement: S1 Fig — (TIF) [file pone.0258765.s002.tif]

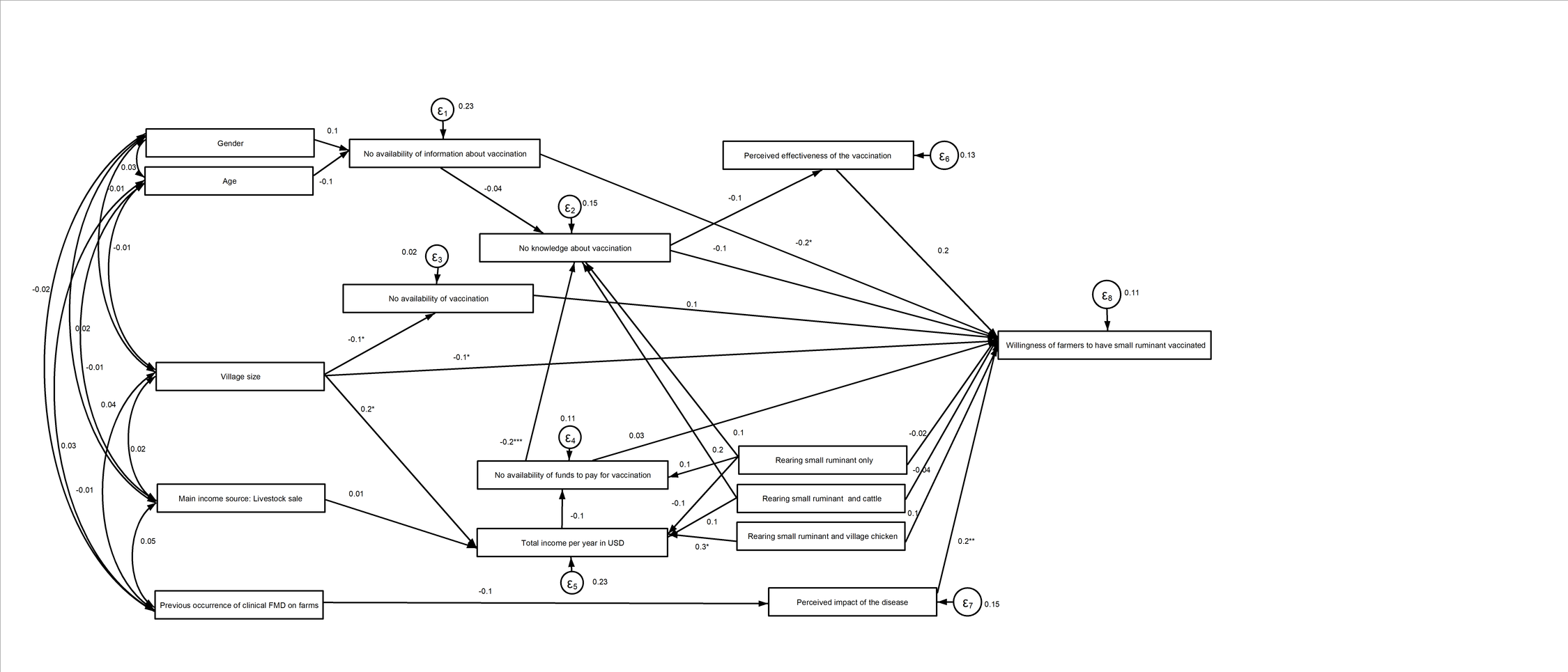

Supplement: S2 Fig — (TIF) [file pone.0258765.s003.tif]

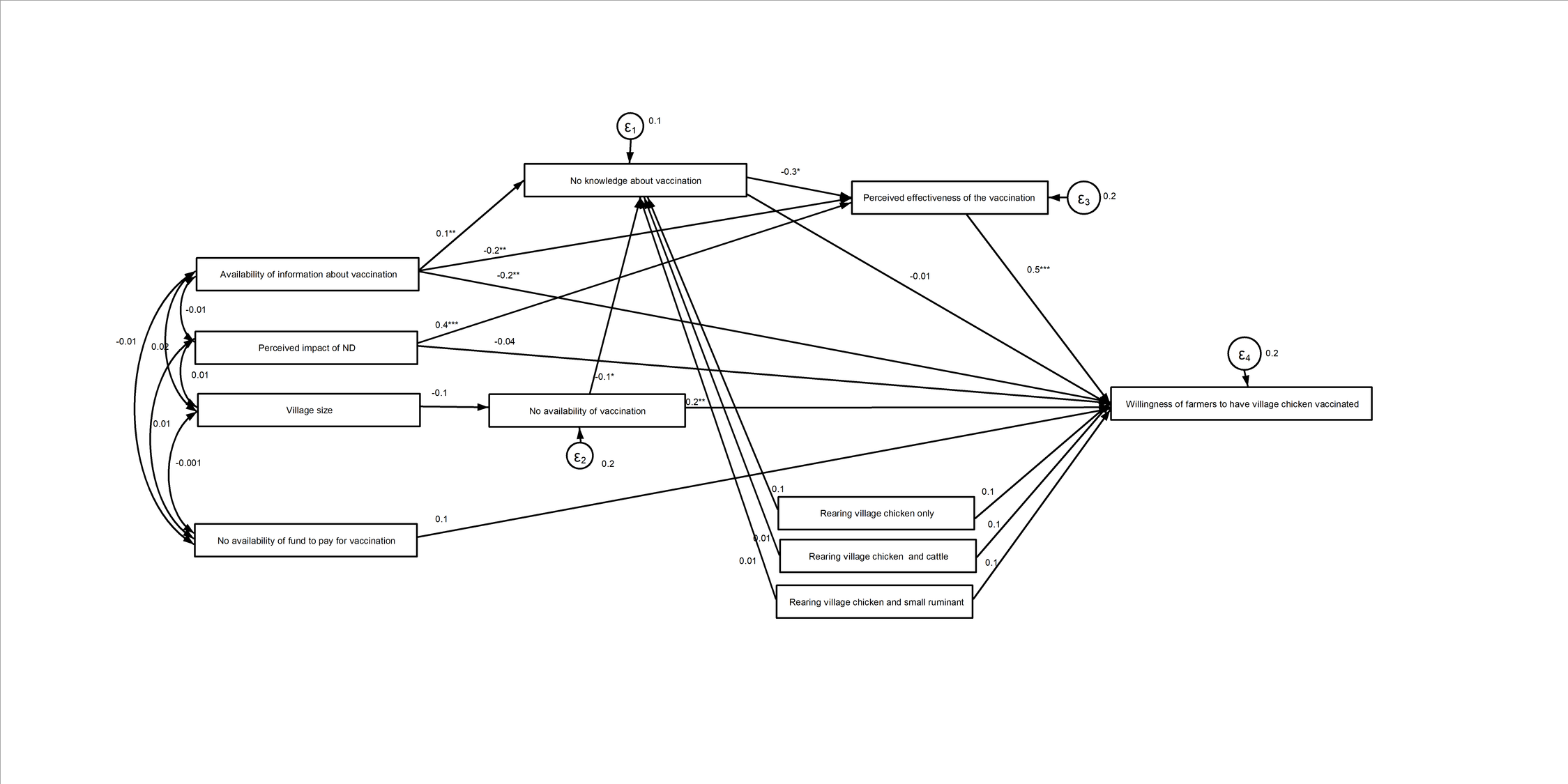

Supplement: S3 Fig — (TIF) [file pone.0258765.s004.tif]
