# Supplementary material for: What drives small-scale farmers to vaccinate their multiple livestock species animals against common infectious diseases in Myanmar?
Source: PLoS One. 2021 Oct 20;16(10):e0258765. doi: 10.1371/journal.pone.0258765 (PMC8528287; doi:10.1371/journal.pone.0258765)
Supplement: S1 Table — (DOCX) [file pone.0258765.s005.docx]

**S1 Table Correlation coefficient of health belief criteria of cattle farmers on FMD vaccination using tetrachoric correlation coefficient** (* *p*<0.05)

**Sub-table 1:**

|  |  | 1 | 2 | 3 | 4 | 5 | 6 | 7 | 8 | 9 | 10 | 11 |
| --- | --- | --- | --- | --- | --- | --- | --- | --- | --- | --- | --- | --- |
| 1 | Perceived impact of FMD | 1.0000 |  |  |  |  |  |  |  |  |  |  |
| 2 | No availability of funds to pay for vaccination | 0.2812 | 1.0000 |  |  |  |  |  |  |  |  |  |
| 3 | No knowledge about vaccination | -0.264 | -1 | 1.0000 |  |  |  |  |  |  |  |  |
| 4 | No availability of vaccination | -0.1295 | **-1.0000*** | **-1.0000*** | 1.0000 |  |  |  |  |  |  |  |
| 5 | Information through farmers | 0.0495 | -0.0196 | -1 | 0.0524 | 1.0000 |  |  |  |  |  |  |
| 6 | Information through local authorities | 0.1215 | 0.1326 | **-0.3788*** | -0.019 | **-1.0000*** | 1.0000 |  |  |  |  |  |
| 7 | Information through traders | 1.0000 | -1.0000 | -1.0000 | -1.0000 | -1.0000 | **-1.0000*** | 1.0000 |  |  |  |  |
| 8 | No information available | **-0.2164*** | -0.1098 | **0.5163*** | 0.0495 | **-1.0000*** | **-1.0000*** | -1.0000 | 1.0000 |  |  |  |
| 9 | Rearing CRL | -0.1004 | -0.0914 | 0.2181 | **-0.2146*** | -0.1407 | 0.1561 | -0.0415 | -0.1222 | 1.0000 |  |  |
| 10 | Rearing CTL + SR | 0.1456 | -0.0117 | 0.0179 | 0.2205 | -0.0345 | -0.2399* | 0.1952 | **0.2565*** | **-1.0000*** | 1.0000 |  |
| 11 | Rearing CTL + CHK | 0.1446 | -0.0691 | -0.243 | 0.0821 | 0.0803 | 0.1243 | -0.2224 | -0.1565 | **-1.0000*** | **-1.0000*** | 1.0000 |
| 12 | Rearing CTL + SR + CHK | -0.1482 | 0.1802 | -0.0372 | -0.0431 | 0.0852 | -0.122 | 0.0851 | 0.0936 | **-1.0000*** | **-1.0000*** | **-1.0000*** |
| 13 | Major income: Livestock sale | 0.0858 | -0.0813 | -0.1378 | 0.124 | 0.1549 | -0.1546 | 0.3018 | 0.0513 | **-0.4164*** | **0.4271*** | -0.147 |
| 14 | Major income: Cropping | 0.0438 | -0.1715 | -0.0696 | 0.0578 | 0.016 | 0.1113 | -0.4362 | -0.0717 | **0.2072*** | **-0.2202*** | -0.0005 |
| 15 | Perceived effectiveness | **0.2348*** | 0.1271 | **-0.4670*** | -0.0882 | 0.1073 | **0.3265*** | 1.0000 | **-0.4322*** | -0.0458 | -0.1576 | 0.2077 |
| 16 | Willingness of farmers to have their animals vaccinated | **0.4242*** | 0.0979 | **-0.7543*** | **0.3290*** | 0.2415 | 0.1688 | -0.0441 | **-0.2590*** | -0.0598 | 0.0233 | 0.0783 |
| 17 | Previous occurrence of clinical FMD on farms | -0.0298 | 0.2214 | -0.0011 | 0.0059 | -0.0348 | 0.0821 | -1.0000 | -0.0312 | -0.0133 | -0.0277 | 0.0156 |
| 18 | Village size | -0.1069 | **0.2922*** | -0.0208 | **0.2096*** | -0.1037 | -0.0319 | -0.2637 | 0.131 | -0.1800* | 0.0801 | -0.0273 |
| 19 | Age | 0.0825 | 0.1683 | -0.0133 | **0.2194*** | **-0.3934*** | **0.2313*** | 0.0431 | -0.118 | 0.0328 | -0.1249 | 0.0058 |
| 20 | Gender | **0.2048*** | -0.1915 | -0.036 | **0.2693*** | -0.0023 | 0.0136 | 0.0253 | -0.0198 | -0.0664 | -0.0309 | 0.0917 |
| 21 | Duration of cattle reared | -0.0424 | -0.2627 | -0.1029 | 0.0635 | -0.0158 | 0.1327 | -0.3434 | -0.0676 | 0.0323 | **-0.2863*** | **0.3069*** |

Sub-table 2:

|  |  | 12 | 13 | 14 | 15 | 16 | 17 | 18 | 19 | 20 | 21 |
| --- | --- | --- | --- | --- | --- | --- | --- | --- | --- | --- | --- |
| 12 | Rearing CTL + SR + CHK | 1.0000 |  |  |  |  |  |  |  |  |  |
| 13 | Major income: Livestock sale | 0.1998 | 1.0000 |  |  |  |  |  |  |  |  |
| 14 | Major income: Cropping | -0.0736 | **-1.0000*** | 1.0000 |  |  |  |  |  |  |  |
| 15 | Perceived effectiveness | -0.0379 | -0.1661 | 0.051 | 1.0000 |  |  |  |  |  |  |
| 16 | Willingness of farmers to have their animals vaccinated | -0.0348 | 0.0386 | 0.0854 | **0.5478*** | 1.0000 |  |  |  |  |  |
| 17 | Previous occurrence of clinical FMD on farms | 0.019 | 0.0063 | -0.0591 | 0.0109 | -0.0289 | 1.0000 |  |  |  |  |
| 18 | Village size | 0.1891 | -0.0621 | 0.0786 | **0.2888*** | **0.2674*** | -0.013 | 1.0000 |  |  |  |
| 19 | Age | 0.0531 | -0.133 | **0.2050*** | **0.2471*** | 0.1524 | -0.1305 | **0.2487*** | 1.0000 |  |  |
| 20 | Gender | -0.0034 | -0.0007 | 0.1486 | 0.1068 | 0.178 | 0.0178 | 0.012 | 0.1584 | 1.0000 |  |
| 21 | Duration of cattle reared | -0.0816 | -0.0336 | **0.3201*** | -0.1964 | 0.1157 | -0.0618 | 0.1405 | 0.1783 | 0.1107 | 1.0000 |
