# Supplementary material for: What drives small-scale farmers to vaccinate their multiple livestock species animals against common infectious diseases in Myanmar?
Source: PLoS One. 2021 Oct 20;16(10):e0258765. doi: 10.1371/journal.pone.0258765 (PMC8528287; doi:10.1371/journal.pone.0258765)
Supplement: S2 Table — (DOCX) [file pone.0258765.s006.docx]

**S2 Table Correlation coefficient of health belief criteria of small ruminant farmers on FMD vaccination using tetrachoric correlation coefficient** (* *p<0.05*)

**Sub-table 1**

|  |  | 1 | 2 | 3 | 4 | 5 | 6 | 7 | 8 | 9 | 10 | 11 |
| --- | --- | --- | --- | --- | --- | --- | --- | --- | --- | --- | --- | --- |
| 1 | Perceived impact of FMD | 1.0000 |  |  |  |  |  |  |  |  |  |  |
| 2 | No availability of funds to pay for vaccination | 0.3645 | 1.0000 |  |  |  |  |  |  |  |  |  |
| 3 | No knowledge about vaccination | -0.2073 | **-1.0000*** | 1.0000 |  |  |  |  |  |  |  |  |
| 4 | No availability of vaccination | -0.0761 | -1.0000 | -1.0000 | 1.0000 |  |  |  |  |  |  |  |
| 5 | Information through farmers | -0.0252 | 0.0252 | -0.1251 | -1.0000 | 1.0000 |  |  |  |  |  |  |
| 6 | Information through local authorities | 0.2349 | **0.3050*** | -0.0828 | 0.1258 | **-1.0000*** | 1.0000 |  |  |  |  |  |
| 7 | Information through traders | 0.1733 | -0.2182 | 0.2328 | -1.0000 | -1.0000 | **-1.0000*** | 1.0000 |  |  |  |  |
| 8 | No information available | **-0.2879*** | -0.2836 | 0.0276 | 0.0262 | **-1.0000*** | **-1.0000*** | **-1.0000*** | 1.0000 |  |  |  |
| 9 | Rearing SR | 0.0335 | **0.2855*** | -0.1022 | 0.0471 | 0.2128 | -0.049 | **0.4782*** | -0.2194 | 1.0000 |  |  |
| 10 | Rearing SR + CTL | 0.0677 | -0.1617 | 0.1723 | -0.1285 | -0.1035 | -0.0265 | -0.3321 | 0.1516 | **-1.0000*** | 1.0000 |  |
| 11 | Rearing SR + CHK | 0.2499 | **-0.4367*** | -0.0572 | -1.0000 | -0.2242 | -0.0681 | -0.102 | 0.1623 | **-1.0000*** | **-1.0000*** | 1.0000 |
| 12 | Rearing SR + CTL + CHK | -0.2304 | 0.0283 | -0.0005 | 0.2282 | -0.03 | 0.1194 | -0.3283 | -0.014 | **-1.0000*** | **-1.0000*** | **-1.0000*** |
| 13 | Major income: Livestock sale | 0.0726 | -0.0926 | -0.1009 | 0.278 | -0.1273 | -0.1903 | 0.2004 | 0.1654 | 0.1538 | 0.0234 | 0.1608 |
| 14 | Major income: Cropping | 0.0345 | 0.1215 | 0.1051 | -0.0858 | -0.042 | 0.2065 | -0.339 | -0.1019 | **-0.2642*** | 0.1477 | **-0.5093*** |
| 15 | Perceived effectiveness | **0.2897*** | 0.1523 | **-0.3681*** | 0.0487 | -0.1282 | **0.2758*** | -0.1727 | -0.1736 | 0.0388 | -0.1345 | -0.0252 |
| 16 | Willingness of farmers to have their animals vaccinated | **0.5340*** | 0.2057 | -0.2561 | 1.0000 | 1.0000 | **0.3101*** | 0.015 | **-0.4017*** | -0.0703 | 0.0524 | 0.2721 |
| 17 | Previous occurrence of clinical FMD on farms | -0.0884 | -0.0584 | -0.1014 | -0.3287 | 0.2715 | -0.1495 | -0.0064 | 0.0712 | 0.1468 | -0.1453 | 0.1103 |
| 18 | Village size | **-0.2477*** | 0.2622 | 0.0491 | 0.425 | -0.3278 | **0.3198*** | **-0.4936*** | -0.0637 | -0.118 | 0.0758 | -0.2035 |
| 19 | Age | -0.0612 | -0.0058 | 0.063 | 0.1919 | -0.2222 | 0.185 | 0.0202 | -0.1384 | -0.0875 | -0.0758 | -0.1905 |
| 20 | Gender | 0.0229 | -0.1966 | 0.1275 | 0.1636 | 0.205 | -0.0317 | -0.2515 | 0.06 | -0.1166 | -0.0046 | -0.0119 |
| 21 | Duration of sheep reared | -0.131 | -0.0519 | **-0.3155*** | **0.7664*** | 0.0571 | 0.1153 | **-1.0000*** | -0.0081 | -0.1567 | -0.0851 | 0.0031 |
| 22 | Duration of goat reared | -0.0612 | **-0.3152*** | **0.2465*** | -0.425 | 0.2362 | -0.1367 | **0.3301*** | -0.0518 | -0.058 | 0.0795 | -0.1014 |

Sub-table 2

|  |  | 12 | 13 | 14 | 15 | 16 | 17 | 18 | 19 | 20 | 21 | 22 |
| --- | --- | --- | --- | --- | --- | --- | --- | --- | --- | --- | --- | --- |
| 12 | Rearing SR + CTL + CHK | 1.0000 |  |  |  |  |  |  |  |  |  |  |
| 13 | Major income: Livestock sale | **-0.2998*** | 1.0000 |  |  |  |  |  |  |  |  |  |
| 14 | Major income: Cropping | **0.4228*** | **-1.0000*** | 1.0000 |  |  |  |  |  |  |  |  |
| 15 | Perceived effectiveness | 0.0923 | -0.083 | 0.0534 | 1.0000 |  |  |  |  |  |  |  |
| 16 | Willingness of farmers to have their animals vaccinated | -0.1214 | 0.111 | -0.0503 | **0.5150*** | 1.0000 |  |  |  |  |  |  |
| 17 | Previous occurrence of clinical FMD on farms | -0.1226 | **0.2402*** | -0.0792 | **0.2875*** | -0.0352 | 1.0000 |  |  |  |  |  |
| 18 | Village size | 0.2076 | -0.1258 | **0.3513*** | 0.2312 | **0.3006*** | **-0.3032*** | 1.0000 |  |  |  |  |
| 19 | Age | **0.2769*** | -0.0995 | **0.2181*** | -0.0491 | -0.1976 | 0.0504 | 0.1812 | 1.0000 |  |  |  |
| 20 | Gender | 0.1327 | 0.1177 | 0.0125 | -0.0996 | 0.1021 | 0.1314 | -0.1222 | **0.2281*** | 1.0000 |  |  |
| 21 | Duration of sheep reared | 0.2101 | 0.03 | **0.2744*** | 0.1821 | 0.109 | **0.3487*** | 0.1049 | **0.2951*** | 0.1733 | 1.0000 |  |
| 22 | Duration of goat reared | 0.0676 | **0.2903*** | -0.1722 | -0.0949 | 0.0108 | 0.1643 | -0.0887 | -0.0736 | -0.0391 | **-0.4799*** | 1.0000 |
