# Supplementary material for: What drives small-scale farmers to vaccinate their multiple livestock species animals against common infectious diseases in Myanmar?
Source: PLoS One. 2021 Oct 20;16(10):e0258765. doi: 10.1371/journal.pone.0258765 (PMC8528287; doi:10.1371/journal.pone.0258765)
Supplement: S3 Table — (DOCX) [file pone.0258765.s007.docx]

**S3 Table Correlation coefficient of health belief criteria of village chicken farmers on ND vaccination using tetrachoric correlation coefficient** (* *p<*0.05)

**Sub-table 1**

|  |  | 1 | 2 | 3 | 4 | 5 | 6 | 7 | 8 | 9 | 10 |
| --- | --- | --- | --- | --- | --- | --- | --- | --- | --- | --- | --- |
| 1 | Perceived impact of FMD |  |  |  |  |  |  |  |  |  |  |
| 2 | No availability of funds to pay for vaccination | 1.0000 | 1.0000 |  |  |  |  |  |  |  |  |
| 3 | No knowledge about vaccination | -0.2135 | -1.0000 | 1.0000 |  |  |  |  |  |  |  |
| 4 | No availability of vaccination | -0.1299 | -1.0000 | -1.0000 | 1.0000 |  |  |  |  |  |  |
| 5 | Information through farmers | 0.0054 | 0.2511 | -1.0000 | 0.1439 | 1.0000 |  |  |  |  |  |
| 6 | Information through local authorities | 0.2058 | 0.1318 | **-1.0000*** | 0.1242 | **-1.0000*** | 1.0000 |  |  |  |  |
| 7 | Information through traders | 1.0000 | -1.0000 | 1.0000 | -1.0000 | -1.0000 | -1.0000 | 1.0000 |  |  |  |
| 8 | No information available | -0.3175 | -0.0593 | **0.3573*** | 0.1952 | **-1.0000*** | **-0.6823*** | -1.0000 | 1.0000 |  |  |
| 9 | Rearing CHK + CTL | 0.0129 | -0.1070 | 0.0253 | -0.1652 | -0.0804 | 0.0160 | -1.0000 | **-0.4989*** | 1.0000 |  |
| 10 | Rearing CHK + SR | 0.1616 | -0.1198 | 0.1040 | -0.0340 | -0.2373 | -0.0029 | 1.0000 | **0.3579*** | **-1.0000*** | 1.0000 |
| 11 | Rearing CHK + CTL +SR | -0.1192 | 0.1895 | -0.1162 | 0.1978 | 0.2217 | -0.0154 | -1.0000 | **0.2610*** | **-1.0000*** | **-1.0000*** |
| 12 | Major income: Livestock sale | -0.2412 | 0.2176 | 0.1162 | -0.1063 | 0.2283 | -0.0777 | 1.0000 | 0.1909 | **-0.4062*** | **0.4197*** |
| 13 | Major income: Cropping | **0.4787*** | -0.0340 | -0.3636 | 0.2246 | 0.0953 | 0.0858 | -1.0000 | -0.1436 | **0.3507*** | **-0.6612*** |
| 14 | Perceived effectiveness | **0.4863*** | 0.0813 | **-0.4815*** | 0.2449 | 0.0067 | 0.2497 | 1.0000 | -0.1952 | 0.0656 | -0.0387 |
| 15 | Willingness of farmers to have their animals vaccinated | **0.3649*** | 0.1262 | **-0.4000*** | 0.2113 | 0.1794 | **0.4234*** | 1.0000 | -0.0520 | -0.0601 | 0.2011 |
| 16 | Previous occurrence of clinical ND on farms | 0.3178 | -0.2760 | 0.0621 | -0.0503 | 0.0621 | 0.1723 | -1.0000 | -0.1139 | -0.0675 | 0.1039 |
| 17 | Village size | -0.1224 | 0.0541 | -0.0760 | 0.1499 | 0.0299 | 0.1532 | -1.0000 | -0.0419 | -0.0560 | -0.2421 |
| 18 | Age | -0.0872 | 0.0111 | -0.0316 | 0.1070 | -0.2431 | 0.0383 | -1.0000 | -0.1081 | 0.1132 | -0.2272 |
| 19 | Gender | **0.5158*** | -0.1107 | -0.2733 | 0.3730* | -0.0633 | 0.1772 | -1.0000 | -0.1627 | 0.1642 | -0.1795 |
| 20 | Duration of village chicken reared | 0.2104 | -0.0709 | -0.0410 | 0.1134 | -0.0410 | 0.1643 | 1.0000 | -0.1060 | 0.2345 | -0.2515 |

**Sub-table 2**

|  |  | 11 | 12 | 13 | 14 | 15 | 16 | 17 | 18 | 19 | 20 |
| --- | --- | --- | --- | --- | --- | --- | --- | --- | --- | --- | --- |
| 11 | Rearing CHK + CTL +SR | 1.0000 |  |  |  |  |  |  |  |  |  |
| 12 | Major income: Livestock sale | 0.0920 | 1.0000 |  |  |  |  |  |  |  |  |
| 13 | Major income: Cropping | 0.0685 | **-1.0000*** | 1.0000 |  |  |  |  |  |  |  |
| 14 | Perceived effectiveness | -0.0422 | -0.1929 | 0.1767 | 1.0000 |  |  |  |  |  |  |
| 15 | Willingness of farmers to have their animals vaccinated | -0.0758 | -0.0905 | 0.2071 | **0.7922*** | 1.0000 |  |  |  |  |  |
| 16 | Previous occurrence of clinical ND on farms | -0.0057 | -0.0362 | -0.1368 | -0.0081 | -0.0022 | 1.0000 |  |  |  |  |
| 17 | Village size | **0.2465*** | **-0.3288*** | **0.3321*** | 0.1506 | 0.2171 | -0.2003 | 1.0000 |  |  |  |
| 18 | Age | 0.0468 | -0.1082 | **0.2657*** | -0.0074 | -0.0424 | **-0.3398*** | **0.2885*** | 1.0000 |  |  |
| 19 | Gender | -0.0453 | -0.0020 | 0.1368 | 0.2312 | 0.2331 | 0.1147 | **0.2343*** | 0.1657 | 1.0000 |  |
| 20 | Duration of village chicken reared | -0.0525 | 0.0248 | 0.1774 | 0.0862 | 0.0310 | 0.0517 | 0.0924 | 0.1510 | **0.3190*** | 1.0000 |
