# Supplementary material for: What drives small-scale farmers to vaccinate their multiple livestock species animals against common infectious diseases in Myanmar?
Source: PLoS One. 2021 Oct 20;16(10):e0258765. doi: 10.1371/journal.pone.0258765 (PMC8528287; doi:10.1371/journal.pone.0258765)
Supplement: S1 File — (PDF) [file pone.0258765.s008.pdf]

<u>Township Survey on Observation of the Animal Production, Animal Health Care System, Trade and Marketing Network in the Central Dry Zone (CDZ) of Myanmar</u>

Survey Objectives

- To observe and describe the traditional animal production system and current animal health care system currently practiced in the Central Dry Zone (CDZ) of Myanmar.
- To observe and describe farmer’s attitude and awareness on animal diseases and major cross-species disease transmission in accordance with the one-health paradigm.
- To observe and describe the animal trade and marketing network in the Central Dry Zone of Myanmar
- To find out the most efficient, reliable and relevant solutions for the development of livestock production and one-health paradigm in the Central Dry Zone of Myanmar by analysing the observations from this survey

Declaration

According to the reports, it is found out that the livestock population is very high in the central part of Myanmar than other regions. Due to the reason of getting little rain in the central part of Myanmar, the central part of Myanmar become named as “Central Dry Zone of Myanmar”, and people, who live in these areas, cannot rely on agriculture and crop production. As a consequence, the animal production become playing a critical role in the Central Dry Zone. Even though the animal production is popular in these areas, the farmers, practicing the animal production in these areas, have still faced with some dilemmas in their animal production such as animal management, animal diseases and trade. The information collected from the farmer will be confidential. The survey is conducted with the purpose of finding out the current animal production system, animal health problem in order to ensure that recommendation for production is useful for famers; and trade and marketing network in the Central Dry Zone of Myanmar, as part of my PhD study.

Information of Survey

1. Date (DD/MM/YY) ____________________
2. Name of interviewer ____________________

Location

3. GPS point ____________________ 4. P-code ____________________
5. Region ____________________
6. District ____________________
7. Township ____________________
8. Village tract ____________________
9. Village ____________________
10. Number of household ____________________

General Information of the Interviewee (Please tick √ the appropriate box)

11. Name - ____________________ 12. Age - ( ) years old
13. Gender - Male ( ) Female ( )
14. Role of the interviewee in the household - (--)
15. Which of the following animal production do you have experience in?
(Please tick √ the appropriate box)

Species	No experience	<5 years	5-10 years	>10 years
Cattle				
Sheep				
Goat				
Village chicken				

16. Which of the following animal production do you rear today?
(Please tick √ the appropriate box)

Species	Rearing today
Cattle	
Sheep	
Goat	
Village chicken	
Other (Describe____________________________)	

Household details

17. Number of family member (Permanent residents) ____________ people
18. Number of family member involving in animal production ____________ people
19. Do you hire labour for animal production? Yes ☐ No ☐

Impact of rearing different types of animal on people income

20. Do you think the regular income from animal production is important for you?

☐ Yes ☐ No

21. Which one is the most important for you and your family regular income?

☐ Animal Production ☐ Crop production ☐ Labour ☐ Other (Describe_________)

22. If animal production is important, what kind of animal do you think you can get more profit?

☐ Cattle production
☐ Sheep production
☐ Goat production
☐ Chicken production
☐ Others (Describe ____)

23. Which of the following factors influence you to decide rearing the following species you have reared? Please give score on that? 0 = Not considerable, 1 = Least considerable, 2 = Moderate considerable, 3 = Highly considerable

Species	*Profit per year*	*Get profit within short period*	*Resistance to disease*	*Easier for trade*	*Multiple purpose*	*Availability of feed*	*Other (Describe____ ____________ _)*	*Total score*
Cattle								
Sheep and Goat								
Village chicken								

24. Are you confident that the animal production can enhance your income?

☐ Yes ☐ No

25. Do you think which of the following production is important for your income?

**Type of animal production**	**Important**	**Moderate**	**Not important**
Cattle production			
Sheep and goat production			
Village chicken production			

26. What could be the main barriers to get regular income from in the following animal production?

**Type of animal production**	**Please specify**
Cattle production	-- --
Sheep and goat production	-- --
Village chicken production	-- --

27. What encourage you to practice the animal production for your income in trading?

**Type of animal**	**Please specify**
Cattle production	-- -- --
Sheep and goat production	-- -- --
Village chicken production	-- -- --

28. Are you confident that the following animal rearing can be a profitable business?

**Type of animal production**	**Yes**	**No**	**Not known**
Cattle production			
Sheep and goat production			

Type of animal production	Yes	No	Not known
Village chicken production			

<u>FMD and ND vaccination practice in cattle, small ruminant and village chicken production</u>

29. Do you know the diseases described below?

Type of animal production	Name of Disease	Yes	No
Cattle production	FMD		
Sheep and goat production	FMD		
Village chicken production	ND		

30. Do you think the incidence of the following disease in your farm animals can cause loss in marketing and trading?

Type of animal production	Name of Disease	Don't know	Yes	No
Cattle production	FMD			
Sheep and goat production	FMD			
Village chicken production	ND			

31. Do you think the vaccination can prevent the following disease occurrence?

Type of animal production	Type of vaccine	Not known	Yes	No
Cattle production	FMD			
Sheep and goat production	FMD			
Village chicken production	ND			

32. If not, what prevention methods are efficient in FMD prevention?

Type of animal	Please specify
Cattle production	--<br>--
Sheep and goat production	--<br>--
Village chicken production	--<br>--

33. Would you like to practice the following vaccination according to your animal species in your farm?

Type of animal production	Type of vaccine	Not known	Yes	No
Cattle production	FMD			
Sheep and goat production	FMD			
Village chicken production	ND			

34. In practicing vaccination, what are the main barriers or obstacles to follow vaccination programme?

Type of animal production	Vaccine	Barrier (Please specify)
Cattle production	FMD	
Sheep and goat production	FMD	
Village chicken production	ND	

35. Where do you get some guidance or instructions about vaccination programme?

Type of animal production	*Vaccine*	*Township vet office*	*Private vet*	*Blue cross worker*	*Middle man*	*Traders*	*Village headman*	*Others*
Cattle production	FMD							
Sheep and goat production	FMD							
Village chicken production	ND							

36. Are you confident that the vaccination will be effective?

Type of animal production	Type of vaccine	Not known	Yes	No
Cattle production	FMD			
Sheep and goat production	FMD			
Village chicken production	ND			
